# Live Imaging of Tumor Initiation in Zebrafish Larvae Reveals a Trophic Role for Leukocyte-Derived PGE_2_

**DOI:** 10.1016/j.cub.2012.05.010

**Published:** 2012-07-10

**Authors:** Yi Feng, Stephen Renshaw, Paul Martin

**Affiliations:** 1 School of Biochemistry and School of Physiology & Pharmacology, Biomedical Sciences Building, University of Bristol, University Walk, Bristol BS8 1TD, UK; 2 MRC Centre for inflammation Research, 47 Little France Crescent, Edinburgh, EH16 4TJ. UK; 3 MRC Centre for Developmental and Biomedical Genetics, The University of Sheffield, Firth Court, Western Bank, Sheffield, S10 2TN, UK

## Abstract

Epidemiology studies and clinical trials have suggested that the use of non-steroidal anti-inflammatory drugs (NSAIDs), including aspirin, can significantly reduce the incidence of and mortality associated with many cancers [1–3], and upregulation of the COX2-PGE_2_ pathway in tumor microenvironments might drive several aspects of cancer progression [4–6]. For these reasons, the mechanisms linking COX blockade and cancer prevention have long been an area of active investigation [7]. During carcinogenesis, COX-2 is expressed both by malignant epithelial cells [8, 9] and by tumor-associated stromal cells, including macrophages [10–12], but the observation that NSAIDs are most effective in cancer prevention in APC^min/+^ mice if the mice are treated from conception [13] suggests that the COX-2/PGE_2_ pathway might also be critical at the earliest stages of tumor development. In this study we take advantage of the translucency and genetic tractability of zebrafish larvae to investigate the involvement of inflammatory cells at cancer initiation, when transformed cells first arise in tissues. We previously showed that innate immune cells supply early transformed cells with proliferative cues [14] and, by using complementary pharmacological and genetic experiments, we now show that prostaglandin E_2_ (PGE_2_) is the trophic signal required for this expansion of transformed cells. Our in vivo observations at these early stages of cancer initiation provide a potential mechanistic explanation for why long-term use of low doses of NSAIDs, including aspirin, might reduce cancer onset.

## Graphical abstract

**Graphical Abstract:**

**Figure:**
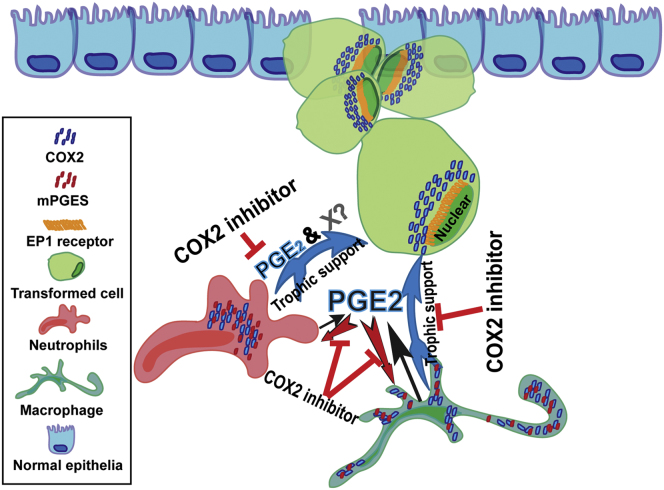

**Highlights:**

►COX-2 inhibition halts early growth of Ras-transformed cells in zebrafish larvae ► Both neutrophils and macrophages contribute to trophic support of transformed cells ► dmPGE_2_ treatment rescues growth of transformed cells after leukocyte depletion ► Inflammation-derived PGE_2_ could be a target for cancer prevention by NSAIDs/aspirin

## Results and Discussion

We have used a human HRAS^G12V^-driven transgenic zebrafish cancer model, in which mucous-secreting cells (analogous to human goblet cells) in the skin are transformed by an eGFP-HRAS^G12V^ fusion protein. Using this model, we have previously shown with a live imaging approach that HRAS^G12V^ (hereafter referred to as V12RAS)-expressing transformed cells induce a robust inflammatory response immediately after their emergence in host skin [14]. Paradoxically, the recruited innate immune cells play a trophic role in promoting the growth of transformed cells [14]. To test whether the COX-2/PGE_2_ pathway might be involved in this process, we decided to block PGE_2_ production.

### Blocking PGE_2_ Synthesis Retards the Growth of Transformed Cells

**Figure 1:**
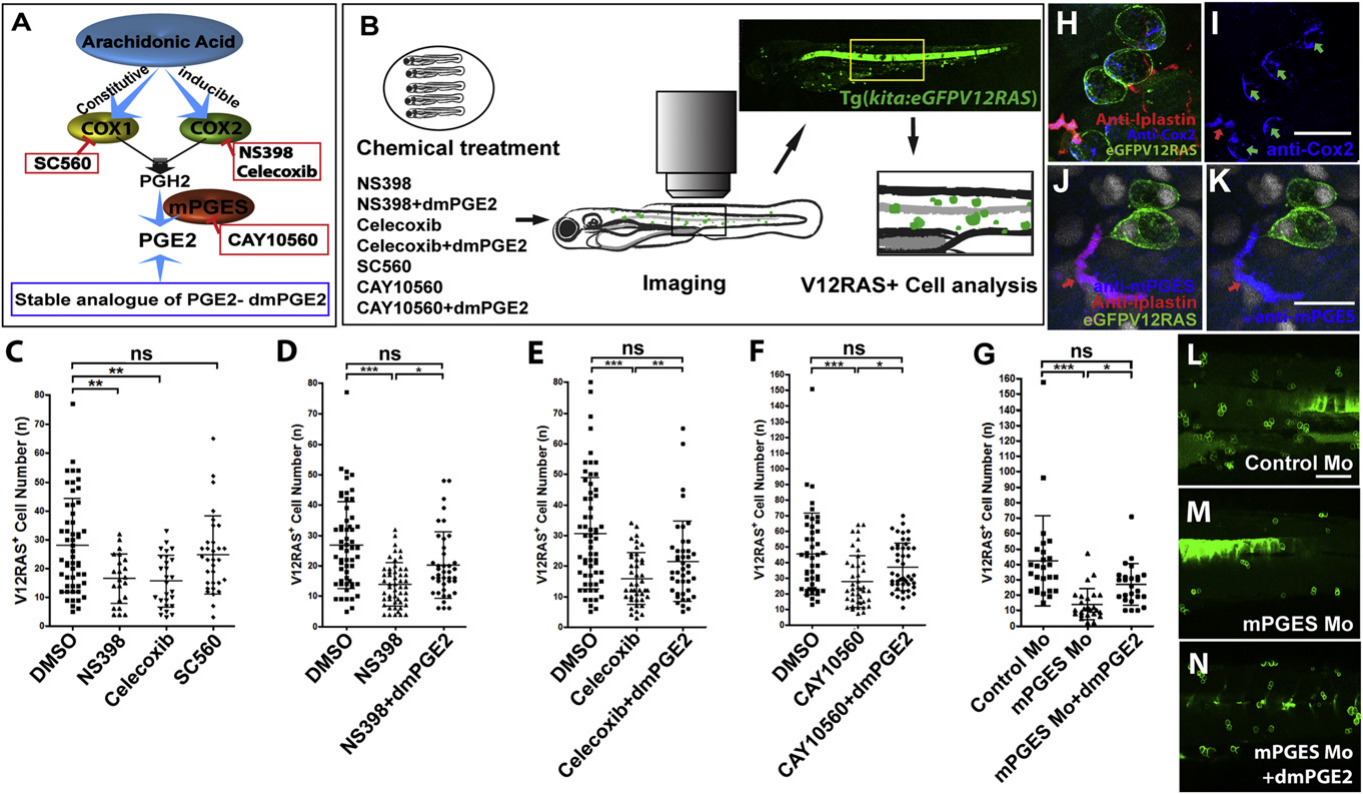
Blocking COX-2-mPGES-Mediated PGE_2_ Production Suppresses the Growth of V12RAS^+^ Transformed Cells In Vivo (A) A schematic representation of PGE_2_ production through the COX-2 pathway indicates the targets and inhibitors used in this study. (B) A schematic representation of our pharmacological treatment regime and clonal analysis of transformed cells (green) in V12RAS^+^-cell-bearing larvae. A yellow box indicates the flank skin region in which we quantified alterations in growth of V12RAS^+^ cells. (C–G) Graphic comparisons of V12RAS^+^ cell numbers in larval flank skin region after various treatments; (C) larvae treated with DMSO, COX-1 inhibitor, SC-560, and the COX-2 inhibitors NS398 and Celecoxib (p < 0.01, n = 22, 27, and 32, respectively); (D) larvae treated with DMSO, NS398, and NS398 +dmPGE2 (p < 0.001, n = 56, 51, and 40, respectively); (E) larvae treated with DMSO, Celecoxib, and Celecoxib +dmPGE2 (p < 0.001, n = 61, 42, and 42, respectively); (F) larvae treated with DMSO, CAY10560 and CAY10560 +dmPGE2 (p < 0.001, n = 45, 42, and 46, respectively); (G) Control morphants, mPGES morphants, and mPGES morphants rescued with dmPGE_2_ (p < 0.001, n = 26, 25, and 25, respectively). (H) Immunostaining for COX-2 (blue) indicates that both leukocytes (anti-L-plastin [red]) and V12RASeGFP^+^ transformed cells (green) express COX-2. (I) Single-channel image of (H), better showing COX-2 expression; green and red arrows indicate transformed cells and leukocytes, respectively. (J) Immunostaining for mPGES (blue) indicates its expression by some leukocytes (anti-L-plastin; red arrow) but not transformed cells (green). (K) Single-channel image of (J). (L–N) Representative images of flank skin regions showing V12RAS^+^ clones (green) of (L) control morphant, (M) mPGES morphant, and (N) mPGES morphant supplemented with dmPGE_2_- Scale bars represent 20 μm (H and I), 15 μm (J and K), and 100 μm (L–N). See also Figures S1, S2, and S3.

PGE_2_ is one of the most abundant PGs produced in the body and plays key roles in mediating the inflammatory response [7, 15]. Cyclooxygenase 1 and 2 (COX-1 and COX-2) catalyze the rate-limiting step in PGE_2_ synthesis by converting arachidonic acid into prostaglandin H_2_, which is subsequently converted to prostaglandins (PGs) via the actions of specific PG synthases (Figure 1A). COX-1 is the housekeeping enzyme that generates PGE_2_ under normal conditions. COX-2 is induced under inflammatory conditions and generates PGE_2_ via mPGES at the site of immune cell recruitment (Figure 1A).

Zebrafish have three COX enzymes, zfCOX-1, zfCOX-2a, and zfCOX2-b [16, 17], and two PGE_2_ synthases, cytosolic PGE_2_ synthase (cPGES) and microsomal PGE_2_ synthase (mPGES) [18]. Because zebrafish larvae are amenable to chemical treatment, we first took a pharmacological approach to suppress PGE_2_ production (as outlined in Figure 1B). Suppression of COX-2 by either NS398 or Celecoxib led to a significant reduction in the number of V12RAS^+^ cells (Figure 1C; Figure S1 in the Supplemental Information available online) without altering expression levels of endogenous *kit a* (the transgene driver) or having adverse effects on larval development (Figure S1). Moreover, ectopically added dmPGE_2_ (a stable analog of PGE_2_) partially restored the number of transformed cells in COX-2-inhibitor-treated larvae (Figures 1D and 1E; Figure S1). No significant reduction in the number of V12RAS^+^ cells was seen in larvae treated with the COX-1 inhibitor SC560 (Figure 1C). These data suggest that in zebrafish, COX-2-mediated production of PGE_2_ promotes the growth of transformed cells.

To establish whether PGE_2_ was indeed the main tumor-promoting mediator generated by COX-2 in our model, we used CAY10560, another inhibitor specific to mPGES, which is known to be downstream of COX-2 in generating PGE_2_ in zebrafish [18]. Again, we saw a reduction in the number of transformed cells; dmPGE_2_ was able to restore the number of transformed cells in combined treatment with CAY10560 (Figure 1F).

### Both V12RAS^+^-Transformed Cells and Recruited Leukocytes Express COX-2, but Only Leukocytes Express mPGES

In mammals, both cancer cells and stromal cells can upregulate COX-2 [8–11], although the contribution of immune-cell-derived PGE_2_ in tumor progression has not yet been defined [5]. We wanted to identify which cell lineages expressed PGE_2_-generating enzymes at the earliest tumor-initiation stage. Double immunostaining showed that both V12RAS^+^ cells (transformed cells) and most L-plastin^+^ cells (neutrophils and macrophages) expressed COX-2 (Figures 1H and 1I). However, we could only detect a signal for mPGES antibody in a subset (approximately 20%) of the recruited leukocytes and in none of the transformed cells (Figures 1J and 1K). These expression profiles imply that, at this early stage, only leukocytes generate PGE_2_.

### Morpholino Knockdown of mPGES Expression Confirms that PGE_2_ Is Required for V12RAS^+^ Overgrowth

Our inhibitor data had demonstrated that COX-2-mPGES-derived PGE_2_ was important for V12RAS^+^ cell growth. To confirm these findings, we used antisense morpholinos to genetically knock down PGE_2_ production. Zebrafish have two genes encoding COX-2 but only one encoding mPGES. Moreover, mPGES only catalyzes PGE_2_ production, and so we used a previously published mPGES morpholino to block its expression [19] (Figure S2). Unfortunately, mPGES-mediated PGE_2_ is required for cell movements during epiboly [19]. To bypass this problem, we rescued normal early development of mPGES morphants by incubating them in dmPGE_2_.Then, after epiboly, we transferred half of the morphants into fresh medium without dmPGE_2_ at 48 hr post-fertilization (hpf; Figure S3); V12RAS^+^ cell numbers were analyzed at 4.5 days post-fertilization (dpf). Again, we saw a reduction in the number of V12RAS^+^ cells in mPGES morphants compared with control-morpholino-injected larvae (Figures 1G, 1L, and 1M), but the number of V12RAS^+^ cells was partially restored in those mPGES morphants that continued with dmPGE_2_ treatment (Figures 1G, 1L, and 1N).

In summary, both our pharmacological inhibition and genetic mPGES knockdown data suggest that PGE_2_ plays an important role in promoting the growth of V12RAS^+^-transformed cells, and our immunostaining data suggest that recruitment of immune cells might be the main source of PGE_2_ at this stage. However, because ectopic dmPGE_2_ failed to completely restore the growth of transformed cells to control levels in either our pharmacological-inhibition or genetic-knockdown experiments, it seems likely that there are also some PGE_2_-independent influences on the growth of transformed cells.

### PGE_2_ Directly Promotes the Growth of Transformed Cells via the EP1 Receptor

**Figure 2:**
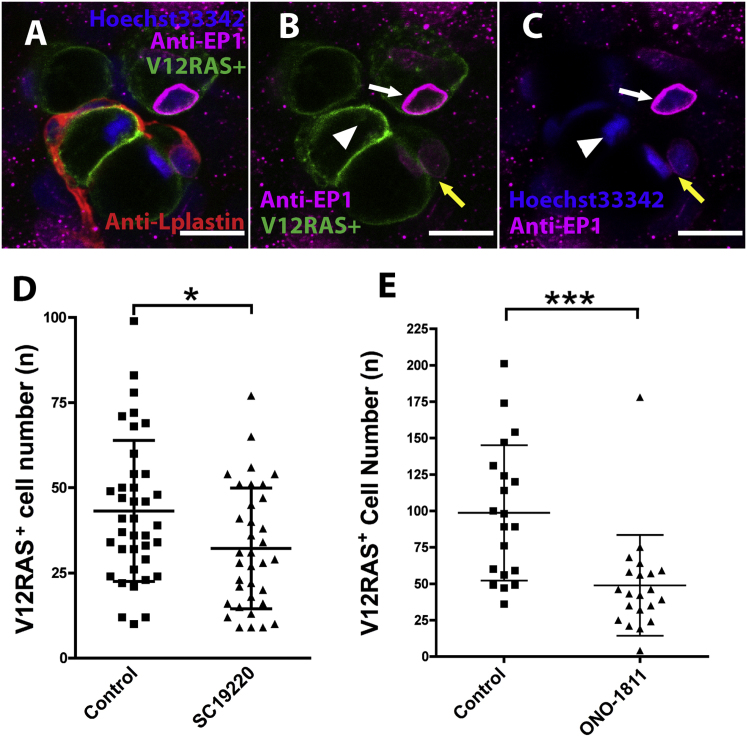
PGE_2_ Promotes Growth of Transformed Cells via the EP1 Receptor (A–C) Immunostaining for EP1 receptor showing EP1 (magenta) localized within V12RASeGFP^+^ cells (green); nuclei are stained with Hoechst 33342 (blue), and leukocytes are revealed by immunostaining with anti-L-plastin antibody (red). EP1 receptor (magenta) shows perinuclear localization (white arrows in [B] and [C]); a pair of recently divided daughter transformed cells exhibit faint EP1 staining (yellow arrows in [B] and [C]), and a transformed cell at metaphase shows no EP1 signal (white arrowhead in [C]). Scale bars represent 10 μm. (D) Graphic illustration showing that EP1 receptor antagonist SC19220 leads to a reduction in the number of transformed cells (p < 0.05, n = 38 and 35). (E) The same is true for the EP1 receptor antagonist ONO-1811 (p < 0,001, n = 20 and 21). See also Figure S1.

To address whether PGE_2_ exerts a direct effect on the growth of transformed cells, we next focused on which of the PGE_2_ receptors was expressed by the transformed cells to mediate such a response. Our immunostaining data indicate that, of the five PGE_2_ receptors that exist in the zebrafish genome (ENSEMBL Zv9), only EP1 is highly expressed by the transformed cells (Figures 2A–2C) and has a particularly pronounced perinuclear localization, as previously reported for EP1 receptor localization in tissue cultured mammalian cells [20]. To test whether EP1 receptor was responsible for mediating the PGE_2_ signal in transformed cells, we used two EP1-specific inhibitors, SC19220 and ONO-8711 (Cayman Chemicals), and both treatments led to a significant reduction in the growth of transformed cells in vivo (Figures 2D and 2E) without adverse effects on larval development (Figure S1). It is thus likely that PGE_2_ promotes the growth of transformed cells through the EP1 receptor.

### An Ectopic Supplement of dmPGE_2_ Can Partially Restore V12RAS^+^ Cell Growth in Leukocyte-Depleted Larvae

**Figure 3:**
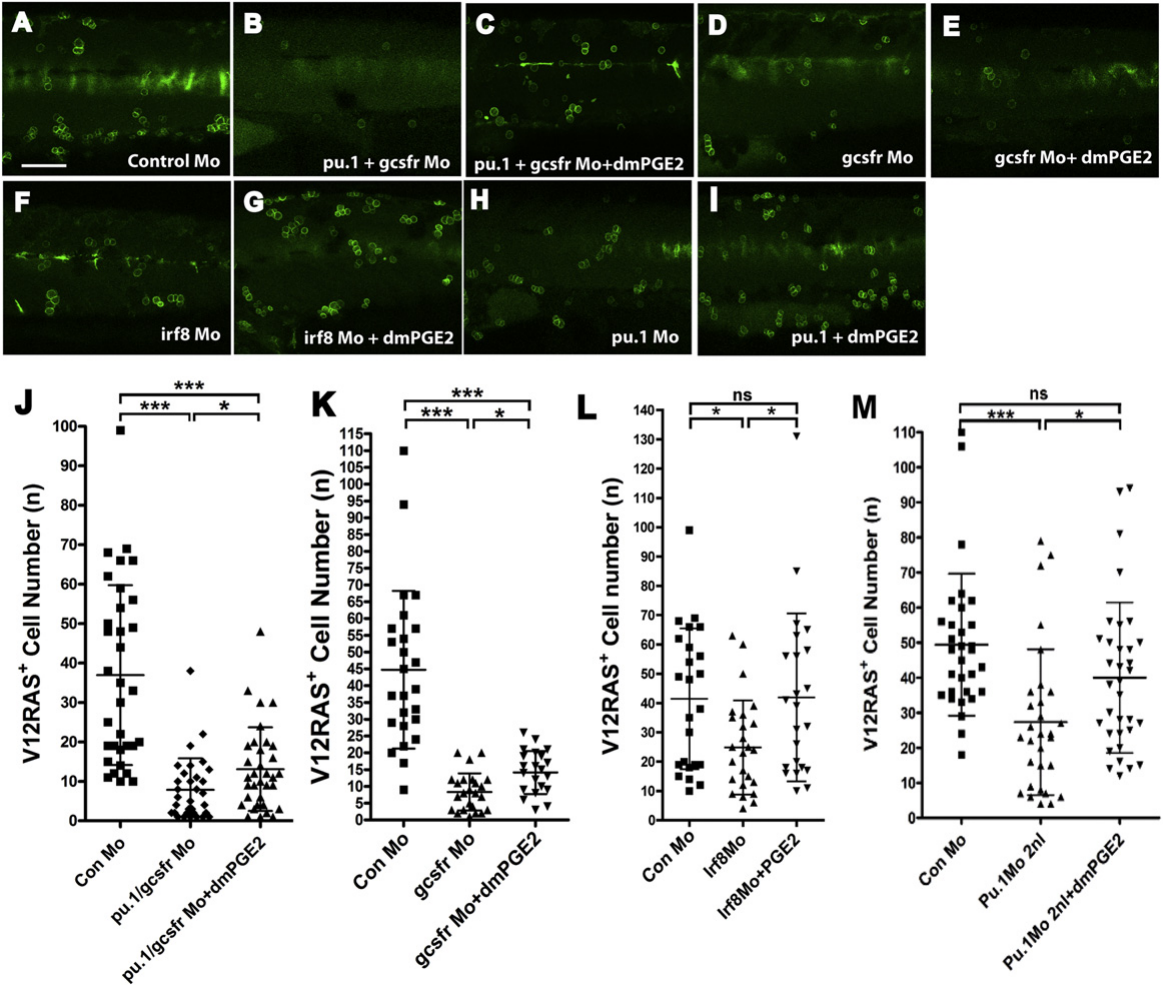
Leukocyte Depletion Prevents V12RAS^+^ Cell Growth, which Can Be Partially Rescued by dmPGE_2_ (A–I) Representative images of flank skin regions illustrating numbers of V12RASeGFP^+^ transformed cells after morpholino (Mo) knockdown of *pu.1* and *gcsfr* (for depletion of both neutrophils and macrophages); *gcsfr* alone (for depletion of neutrophils); *irf8* or a lower dose (2 nl/embryo) of *pu.1* morpholino alone (for depletion of macrophages) with or without additional treatment with dmPGE_2._ (J) Graphic illustration of how dmPGE_2_ can rescue the number of transformed cells in morphants in which both neutrophils and macrophages have been specifically depleted (p < 0.01, n = 33, 32, and 34). (K) Graphic illustration of how dmPGE_2_ can only partially rescue morpholino knockdowns that largely target neutrophils (p < 0.001, n = 25, 25, and 22). (L and M) Similar graphic illustration of how dmPGE_2_ can rescue morpholino knockdowns that largely target macrophages (irf8 Mo—p < 0.05, n = 24, 26, and 23; 2 nl pu.1 Mo—p < 0.05, n = 31, 30, and 34). The scale bar represents 100 μm. See also Figures S3 and S4.

Our data herein suggest that PGE_2_ is produced by leukocytes as trophic support for the growth of transformed cells. If this is indeed the case, then we should be able to restore tumor growth in leukocyte-depleted larvae by adding PGE_2_. We thus blocked leukocyte development by using a combined morpholino *pu.1+gcsfr* double knockdown, which effectively arrests all myeloid lineage development in zebrafish larvae until at least 4 dpf [21]. Leukocyte depletion does not affect general embryo development (Figure S4A–S4G); however, this absence of leukocytes led to a dramatic reduction in the number of V12RAS^+^ cells (Figures 3B and 3J). When we supplied dmPGE_2_ to the leukocyte-depleted double morphants, we saw a partial restoration of V12RAS^+^ cell numbers (Figures 3C and 3J). These data suggest that immune cell trophic support is mediated through the PGE_2_-EP1 receptor pathway, which acts directly on the transformed cells.

### dmPGE_2_ Fully Restores V12RAS^+^ Cell Numbers in Macrophage-Depleted Larvae but Only Partially Does So in Neutrophil-Deficient Larvae

In our previous studies, we found that the transformed cells trigger recruitment of several innate immune cell lineages [14]. We now wished to dissect the contribution of individual leukocyte lineages to the growth of transformed cells. In zebrafish, neutrophil development is severely impaired in *gcsfr*-morpholino-injected larvae, whereas macrophage numbers are only slightly decreased [21] (Figure S4K). In such neutrophil-depleted morphants, we observed that V12RAS^+^ cell numbers are reduced almost to the numbers seen in *pu.1*+*gcsfr* morphants (which lack both neutrophils and macrophages; Figures 3B and 3D; Figure S4I). This suggests an important trophic role for neutrophils during the initial expansion of transformed cells.

IRF8 is a transcription factor that regulates the differentiation of a common progenitor into macrophages rather than neutrophils, and *irf8* morpholino treatment depletes macrophages and results in a compensatory increase in neutrophils [22] (see Figure S4J). In these macrophage-depleted larvae, we again saw a reduction in the number of V12RAS^+^ cells (reduced to 41.9%) but to a much lesser degree than in total leukocyte (*pu1+gcsfr* morphant, macrophage + neutrophil)-depleted larvae (reduced to 21.1%) (Figures 3B, 3F, and 3J). We confirmed that macrophage depletion alone (i.e., without a compensatory increase in neutrophils) also leads to a reduction in the number of V12RAS^+^ cells number (reduced to 49%) by using low-dose *pu.1* morpholino (Figures 3H and 3L). These data suggest that macrophages contribute to the trophic support of transformed cells but to a lesser extent than neutrophils.

Moreover, the nature of trophic support received from neutrophils could be qualitatively different from that received from macrophages. When dmPGE_2_ was used to treat larvae that had been depleted of different immune cell types, we saw differences in the rescue profile of transformed-cell growth. In both *pu.1*+*gcsfr* double morphants (lacking both macrophages and neutrophils) and *gcsfr* morphants (lacking only neutrophils), dmPGE_2_ only partially restored V12RAS^+^ cell numbers (Figures 3C, 3E, 3J, and 3K). However, in macrophage-depleted *irf8* morphants and low-dose *pu.1* morphants, dmPGE_2_ treatment completely restored the number of transformed cells (Figures 3G, 3I, 3L, and 3M). It has previously been shown that PGE_2_ can drive hematopoietic stem cell expansion [23], and so we tested whether leukocyte rescue by dmPGE_2_ treatment might indirectly cause increased numbers of transformed cells, but we saw no significant recovery of depleted innate immune cells in these larvae (Figures S4I–S4N). These findings suggest that macrophages contribute PGE_2_ alone, whereas neutrophils might produce other trophic factor(s).

### Reduced PGE_2_ Production Leads to Altered Neutrophil Migration in the Vicinity of Transformed Cells, and Increased Cell Death of Transformed Cells Follows along with Their Rapid Engulfment by Macrophages

It has been shown that PGE_2_ regulates immune cell function, and several recent reports suggest that PGE_2_ might induce an immunosuppressive phenotype in leukocytes [24–26]. We wondered whether suppressing PGE_2_ production with COX-2 inhibitors might modulate the early inflammatory response and change the way in which leukocytes and transformed cells interact.

**Figure 4:**
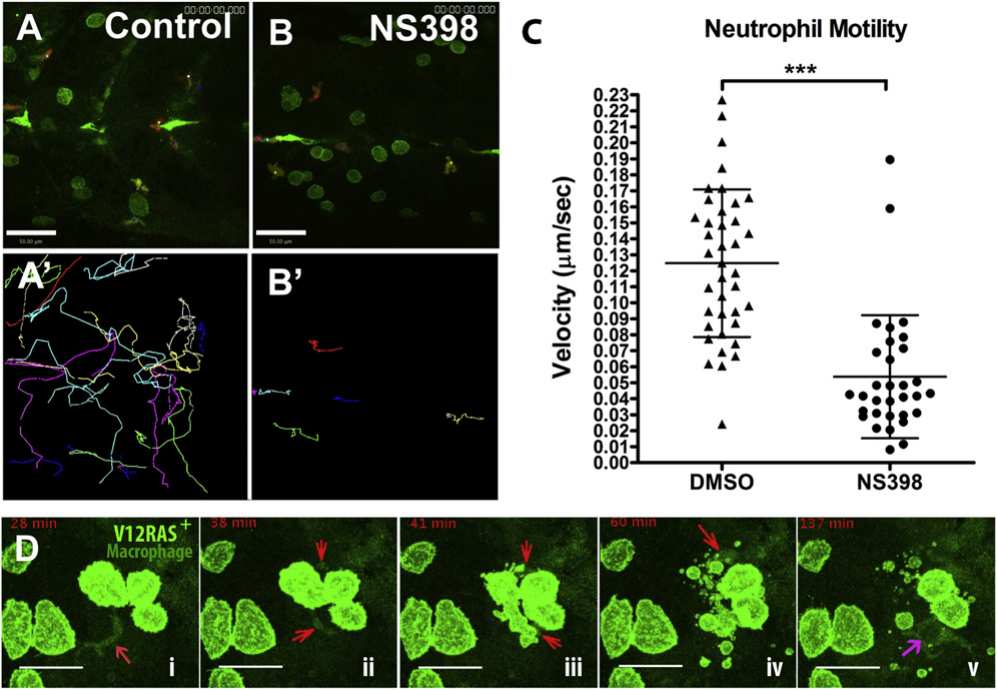
Live Imaging Reveals that Reduced PGE_2_ Levels as a Result of COX-2 Inhibition Lead to Leukocyte Behavior Change and Increased Transformed-Cell Death (A and B) Still images from movies illustrating neutrophil (red) numbers and their motility in the vicinity of V12RAS transformed cells (green) in (A) control and (B) NS398-treated larvae (images are taken from Movie S1) (A′ and B′) show the tracks over 100 min of neutrophil movements from the two movies S1A and S1B, respectively, revealing reduced neutrophil motility in NS398-treated (B′) compared with control (A′) larva. Scale bars represent 50 μm. (C) Quantification of neutrophil migration velocity (V = mean ± SD); DMSO-treated V = 0.125 ± 0.046 μm (n = 39); NS398-treated V = 0.054 ± 0.038 μm (n = 32). (D) A series of still images from a time-lapse movie (see Movie S2) showing transformed-cell death associated with a macrophage encounter in a larva treated with NS398; subsequently, this macrophage and others engulf the resulting cell debris. Red arrows in (i)–(iv) indicate a macrophage labeled by NFκB-RE:eGFP (pale green). Purple arrow in (v) indicates a macrophage engulfing the transformed-cell debris and forming a phagosome inside its cell body. The scale bar represents 15 μm.

Cell-tracking analysis of time-lapse movies show that in COX-2 inhibitor (NS398)-treated larvae, neutrophils near clones of transformed cells show considerably reduced motility (migration velocity drops from 0.125 ± 0.046 μm to 0.054 ± 0.038 μm, [mean ± SD]) (Figures 4A, 4A′, 4B, 4B′, and 4C; also see Movie S1) in comparison to those in untreated transformed-cell-bearing larvae, indicating that reduced PGE_2_ does indeed lead to a change in neutrophil behavior.

Live-imaging studies by us and others have defined the “inflammatory macrophage” in zebrafish larvae by their typical elongated, dendritic morphology and slow patrolling behavior [14, 27]. Here we used *Tg* (*NFκB-RE:eGFP; LysC:DsRed; kit a:GalTA4;UAS:eGFPV12RAS*) larvae, in which macrophages are labeled with low levels of NFκb reporter GFP (Figure 4D; also see Movie S2) to reveal their behavior near clones of transformed cells. In untreated larvae, transformed-cell deaths are sufficiently rare that we have never captured them by live imaging in either the presence or the absence of immune cells [14]. By contrast, in larvae treated with COX-2 inhibitor (NS398), we frequently saw transformed-cell deaths, and often these were associated with prior macrophage encounters (Figure 4D; see also Movie S2). The corpses of transformed cells were subsequently rapidly cleared away by adjacent macrophages. These data suggest that reduction of PGE_2_ levels by COX-2 inhibition leads to a change of macrophage behavior and enhanced proinflammatory activity, which results in a more active engagement and engulfment of transformed cells. This is consistent with recent reports that PGE_2_ directs macrophages toward a tumor-promoting M2 phenotype [24, 28] and can redirect dendritic cells toward a stable myeloid-derived-suppressor-cell phenotype [25].

Our previous and current studies reveal a pivotal contribution of host immune cells to the optimal growth of transformed cells as they first arise in tissues [14]. Here we show that leukocyte-derived PGE_2_ is a key trophic factor for transformed cells at these early stages of cancer initiation. Our data suggest that PGE_2_ might work in two complementary ways, both directly on transformed cells via the EP1 receptor and indirectly by promoting a “trophic inflammatory cell” behavior in neutrophils and macrophages; this behavior might also support the survival of transformed cells by inhibiting killing and engulfment.

We have provided evidence here that a trophic inflammatory response is important for a transformed cell to grow at its inception and that PGE_2_ produced by innate immune cells via the COX-2 pathway is a key trophic factor for optimal growth of transformed cells at the earliest stages of tumor progression. Moreover, this trophic inflammatory response can be suppressed by the inhibition of PGE_2_ production via COX-2 inhibitors, which might explain why use of non-steroidal anti-inflammatory drugs (NSAIDs) can reduce cancer incidence.

## Experimental Procedures

### Zebrafish Strains and Maintenance

Adult zebrafish (*Danio rerio*) were maintained as previously described [29]. All experiments were conducted with local ethical approval from the University of Bristol and in accordance with UK Home Office regulations. Strains included *Tg(5XUAS:eGFP-H-RASV12)io6* [30], *Et*(*kit a:GalTA4,UAS:mCherry*)*hzm1* [30], *Tg*(*LysC:DsRed*)*nz* [31]; the *Tg(NFκB-RE:eGFP)i235* line was generated with the method and constructs described in Kanther et al. [32].

### Morpholino Injection

All morpholinos were obtained from (GeneTools LLC, Philomath, OR, USA) and injected into one-cell-stage embryos as previously described [19, 21, 22, 33]. For sequence information, see Supplemental Experimental Procedures.

### Immunofluorescence, Live Imaging, and Tracking of Cell Movements in Larvae

Whole-mount immunostaining was performed as previously described [14]. For all our live-imaging studies, time-lapse movies were collected with a Leica SP5-AOBS Confocal Laser-Scanning Microscope. An Image J 1.4, Manual Tracking plugin was used for cell tracking (further details are available in the Supplemental Experimental Procedures).

### Pharmacological Treatment and Clonal Analysis of the Progression of Transformed Cells

Transformed-cell-bearing larvae were treated with inhibitors of PGE_2_-synthesis enzymes or dmPGE_2_ from 48 hpf to 88 hpf or from 48 hpf to 96 hpf in 0.3% Danieau's solution containing 1% DMSO. All the inhibitors were from Cayman Chemicals. For detailed procedures, see Supplemental Experimental Procedures.

### Statistics

All the data were analyzed (Prism 4.1, GraphPad Software, La Jolla, CA, USA) with an unpaired two-tailed Student's t test for comparisons between two groups with a one-way ANOVA and appropriate post-test adjustment for comparisons of multiple groups.

## Supplemental Information

Document S1. Figures S1–S4 and Supplemental Experimental Procedures

Movie S1. Supporting Movies for Figures 4A and 4B Movies from the flank skin region of a *Tg(kit a:GalTA4, UAS:V12RASeGFP, lysC:DsRed)* larva at 3 dpf. Green cells are V12RAS^+^ transformed mucus-secreting cells; red cells are LysC:DsRed labeled neutrophils. On the left is a typical 3 dpf control larva where we see LysC:DsRed^+^ leukocytes actively patrolling through the territory occupied by transformed cells. On the right is a typical movie of an NS398-treated larva showing significantly reduced neutrophil patrolling velocity at 3 dpf.

Movie S2. Supporting Movie for Figure 4D Movie from the flank skin region of a typical *Tg(kit a:GalTA4, UAS:V12RASeGFP*, *NFκB-RE*:eGFP) larva treated with NS398. Rounded bright green cells are V12RAS^+^ transformed mucus-secreting cells, and elongated pale green cells are patrolling macrophages. A red arrow indicates an early macrophage (blue dot) as it arrives in the vicinity of the clone of transformed cells and makes initial direct contacts with one of the cells in the group. Soon afterward, the transformed cell undergoes a catastrophic cell death, and the first macrophage as well as others (purple arrow, red dot) are seen engulfing debris resulting from this death.
