# Supplementary material for: Live Imaging of Tumor Initiation in Zebrafish Larvae Reveals a Trophic Role for Leukocyte-Derived PGE2
Source: Curr Biol. 2012 Jul 10;22(13):1253–9. doi: 10.1016/j.cub.2012.05.010 (PMC3398414; doi:10.1016/j.cub.2012.05.010)
Supplement: Document S1. Figures S1–S4 and Supplemental Experimental Procedures [file mmc1.pdf]

Current Biology Volume *22*

Supplemental Information

Live Imaging of Tumor Initiation in Zebrafish Larvae Reveals a Trophic Role for Leukocyte-Derived PGE_2

Yi Feng, Stephen Renshaw, and Paul Martin

Figure S 1

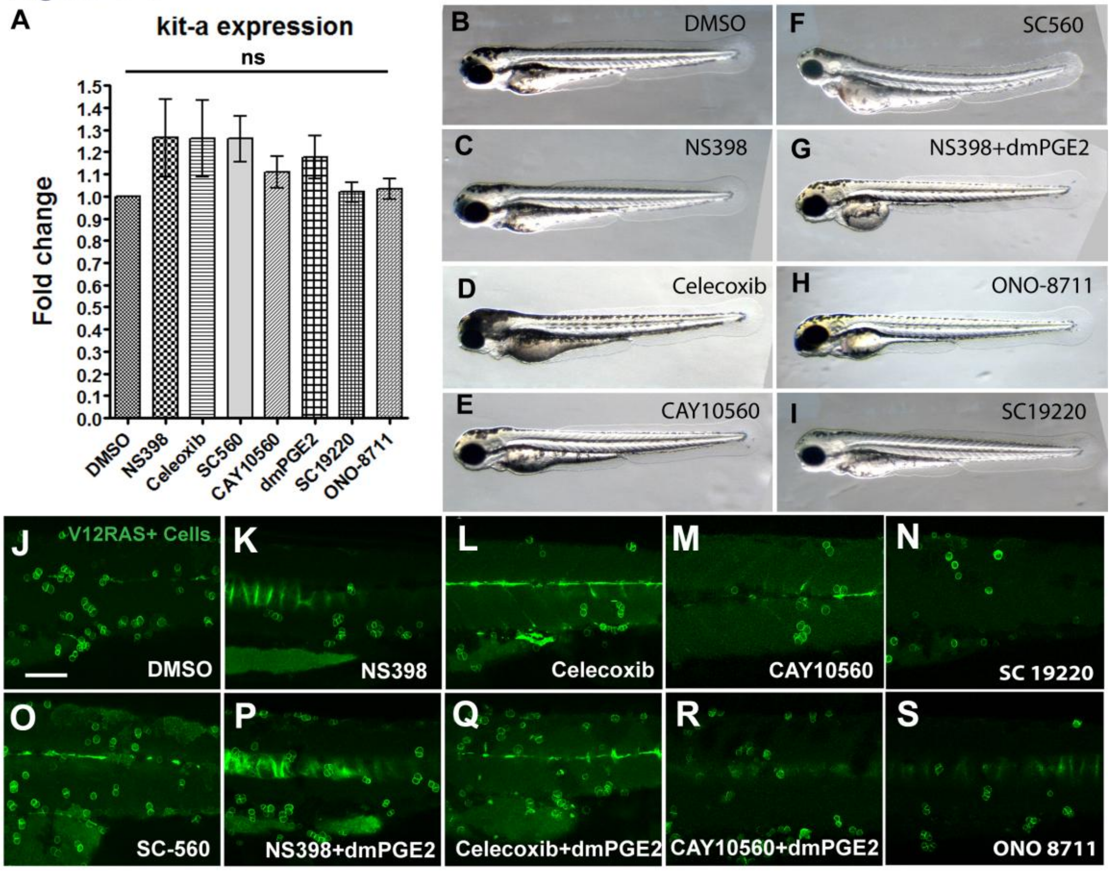

Figure S1. Chemical treatments do not affect endogenous kit-a expression or larval development (related to Figures 1 and 2).

(A) q-PCR data for kit-a expression by chemical-treated larvae confirming that these chemical treatments do not alter transgene expression level. (B-I) Wide field images of chemical-treated larvae at 4dpf, showing that our various chemical treatments does not result in any obvious developmental abnormalities. (J-S) Representative images of flank skin regions showing V12RAS$^+$ clones (green) of (J) DMSO, (K) NS398, (L) Celecoxib, (M) CAY10560, (N) SC19220, (O) SC-560, (P) NS398+dmPGE$_2$ (Q) Celecoxib+dmPGE$_2$, (R) CAY10560+dmPGE$_2$, (S) ONO-1811. Scale bars in J-S = 100μm.

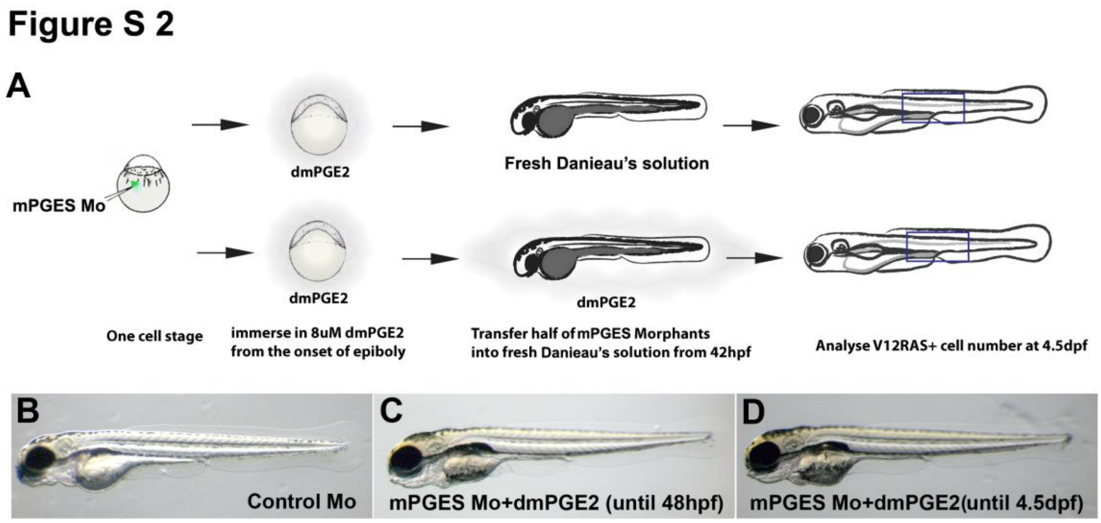

Figure S2. Schematic to illustrate mPGES morpholino experiment + larval images to show the normal development of treated larvae (related to Figure 1).

(A) Schematic outline of our procedure for morpholino knockdown of mPGES expression in zebrafish larvae. In both treated groups the medium was supplemented with stable PGE_2 ($dmPGE_2$) to compensate for the PGE_2 required for normal development up to 42hpf; subsequently, $dmPGE_2$ was removed from the PGE_2 suppressed group. (B-D) Wide field images of the morphologies of Control morphant (B), mPGES Morphant with dmPGE2 rescued until 48hpf (C), and mPGES Morphant larvae with dmPGE2 rescued until 4.5dpf (D).

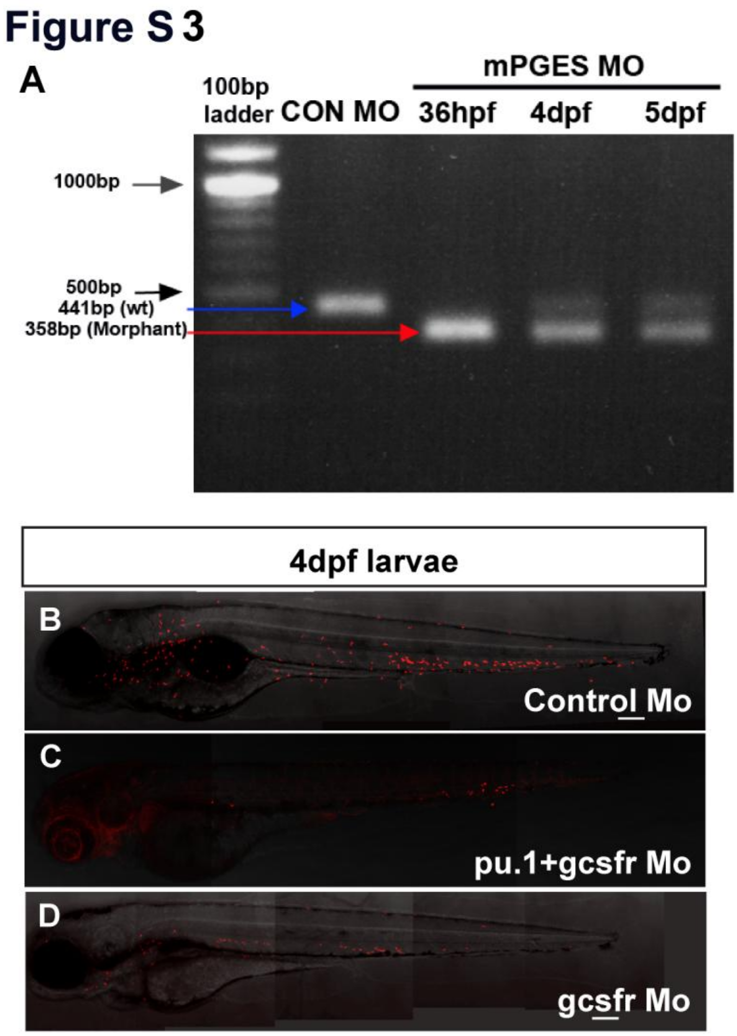

Figure S3. Morpholinos are still effective throughout the timecourse of our transformed cell number analysis (related to Figures 1 and 3)

(A) RT-PCR results showing that mPGES morpholino is still capable of significant knockdown of wild type mPGES mRNA until at least 5dpf. (B-E) Wide field images overlayed with red fluorescent views of Tg (*lysC:DsRed*) larvae, showing neutrophils still suppressed in pu.1+gcsfr Morpholino and gcsfr Morpholino injected larvae at 4.5dpf. Scale bar=150µm.

Figure S 4

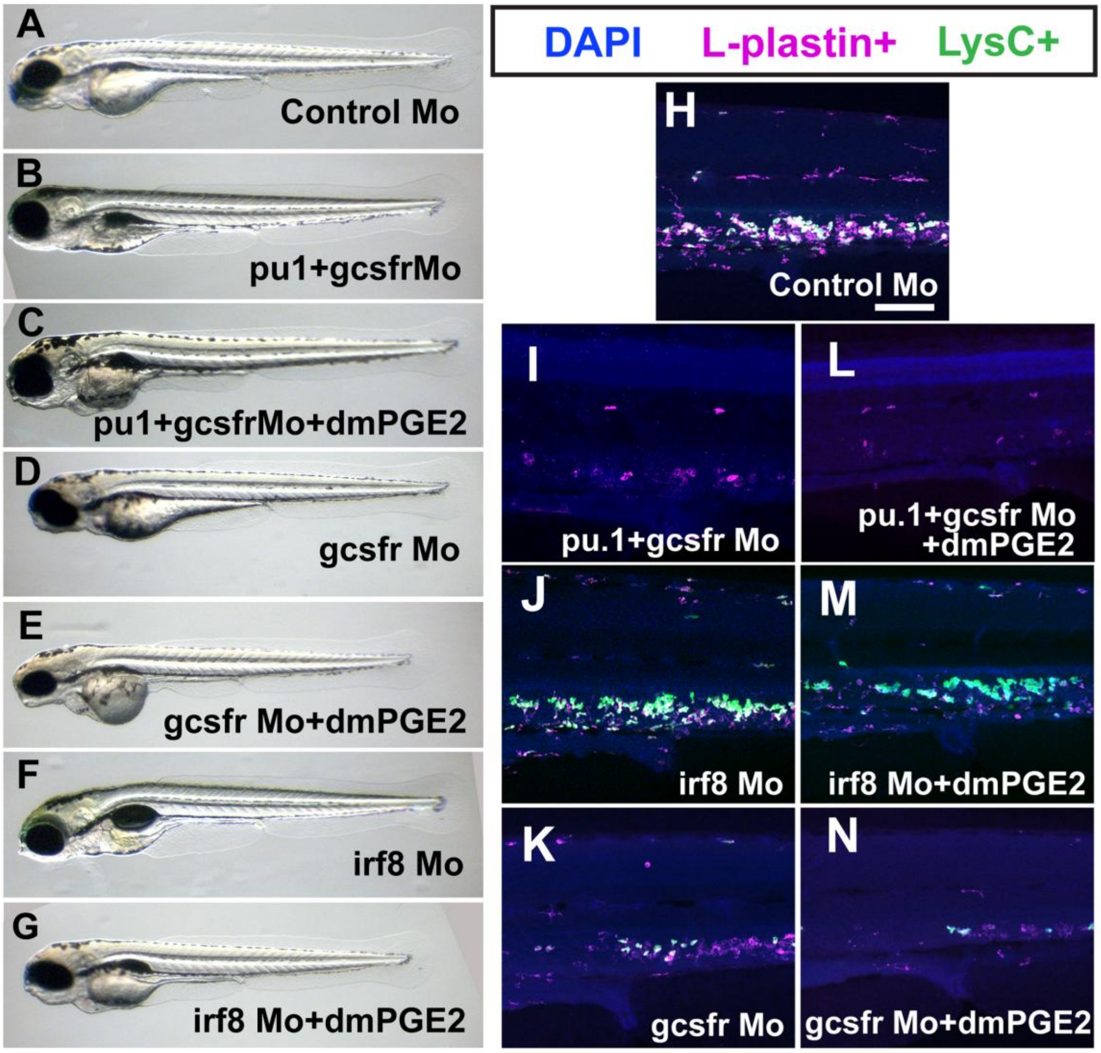

Figure S4. Normal morphology of leukocyte-depleted morphants (related to Figure 3)

(A-G) Wide field images of morphants showing no general morphological defects; (H-N) Confocal images of anti-L-plastin immunostaining (magenta) of Lys:DsRed$^+$ (green) larvae, showing no increase of L-plastin$^+$ cells (magenta) or LysC:DsRed$^+$ cells (green) in dmPGE$_2$ treated, pu1+gcsfr morphants (L), irf8 morphant (M) or gcsfr morphants (N), when compared with untreated equivalent morphants (I, J, K respectively). Scale bar=100μm.

Supplemental Experimental Procedures

Pharmacological treatment and clonal analysis of transformed cell progression

Transformed-cell bearing larvae were treated with inhibitors of PGE_2 synthesis enzymes or $dmPGE_2$ from 48hpf – 88hpf or 48hpf – 96hpf in 0.3% Danieau's solution containing 1% DMSO. All the inhibitors were from Cayman Chemicals. COX-2 inhibitor NS398 30μM; and Celeoxib were used at 25μM; COX-1 inhibitor SC560 was used at 15μM; mPGES inhibitor CAY10560 was used at 10μM; EP1 antagonist ONO-8711 was used at 10μM; mPGES, *pu.1, pu.1*+*gcsfr* and *irf8* morphants were either treated with 1% DMSO as control or 1%DMSO containing 10μM $dmPGE_2$. After treatment, larvae were fixed in 4% PFA for 1 hour at room temperature, rinsed in PBS and lateral mounted, then imaged using a Leica Confocal SP5 with a 20X glycerol lens at 1.5 Zoom, to count numbers of $V12RASeGFP^+$ cells in a standard flank skin area. All the inhibitor experiments were repeated at least 5 times. Data from three independent experiments were pooled and then subjected to statistical analysis.

Morpholino and PCR primer sequences used in this study

Previously described morpholinos including mPGES Mo (5′-GTTTTGTGCTCTTACCTCCTACAGC-3′) [1] *pu.1* Mo (5′-GATATACTGATACTCCATTGGTGGT-3′) [2]; *gcsfr* Mo 5′-GAAGCACAAGCGAGACGGATGCCAT-3′ [3]; *irf8* Mo 5′-AATGTTTCGCTTACTTTGAAAATGG-3′ [4] and standard control morpholinos were injected into one cell stage embryos as previously described [1-4].

Zebrafish mPGES (Gene Name: prostaglandin E synthase) ZFIN ID: ZDB- GENE-050407-2; Enzembl transcript ID: OTTDART00000050114

Primers for amplifying full-length sequence to check morpholino efficiency

zfmPGES-N: 5'-ATGCTCGGGAGCGACATACA-3'; zfmPGES-C: 5'-TCATGCGAATGAGGCCACTT-3';

mPGES splicing block Morpholino (sequence: 5' - GTTTTGTGCTCTTACCTCCTACAGC - 3' [1]) is target to the E2-I2 boundary; the targeted reverse complementary sequence is shown below: Exon2-(5'GCTGTAGGAG)-Intron2(gtaagagcacaaaac3')

This morpholino leads to exclusion of the Exon 2 (83bp), leading to a shortened morphant cDNA product (358bp) compared with the WT cDNA product (441) (figure S 3).

Zebrafish kit-a Quantitative PCR

Larvae were treated with various chemicals as described in Experimental procedures, at the end of treatment 10 larvae from each treatment group were collected in Qiazol® (Qiagen) and total RNA was extracted following the manufacturer's protocol. cDNAs were synthesized using a SuperScript® VILO™ cDNA Synthesis Kit (Invitrogen) following manufacturer's protocol. 2 μg of total RNA was used in a 20ul RT reaction for each sample. Quantitative PCR reactions were performed using Option Monitor 3.1.32 (Bio-Rad Laboratories, Inc). Cycle parameter: 95 degree 10 minutes followed by 95 degree 15 seconds 60 degree 30 seconds for 40 cycles. *kit-a* expression levels were normalized with internal control *zfEFα*. Zebrafish *kit-a* q-PCR primers: *zfkit-a*F 5'-TCCTGATCGTGCTGACCTAC-3'; *zfkit-a*R 5'-GTATGGAAGCTGAGTGGGGT-3'; Internal control zebrafish Elongation factor alpha (EFα) primer squences: *zfef*αF 5'-CTGGTTCAAGGGATGGAAGA-3', *zfef*αR 5'-GAGACTCGTGGTGCATCTCA-3'; Results were shown as fold change of *kit-a* expression of chemical-treated larvae compared with vehicle (DMSO)-treated control larvae (figure S1 A). Q-PCR analysis of kit-a expression was performed for three independent treatment experiments and one way ANOVA was used for statistical analysis which showed no significant differences in kit-a expression between control and chemical treated larvae.

Wholemount Immunofluorescence

Embryos/larvae were fixed in 4% PFA+0.4% Triton X-100 at room temperature for 2hrs prior to rinsing, blocking and incubation with primary antibodies overnight at 4-°C. Primary antibodies used in this study include rabbit polyclonal, anti-L-plastin antibody (1:500), goat anti-COX-2 (1:100) (Cat 100034, Cayman Chemical, MI USA), rabbit anti-mPGES (1:100) (Cat. 160140, Cayman Chemical, MI USA), rabbit anti-EP1 (1:100) (Cat. 101740, Cayman Chemical, MI USA). Subsequently, either Cy3 or Cy5 conjugated secondary antibodies (Jackson Lb, USA) was used to reveal primary antibody localization.

Live imaging and time-lapse imaging of zebrafish embryos

For all of our live imaging studies, larvae were mounted on their sides in 1.5% low melting Agarose (Sigma, UK), in a glass bottomed dish, filled with 0.3% Danieau's solution containing 0.01 mg/ml Tricaine. The climate chamber covering the microscope stage was set at 27.5 ^{o}C. Images were collected using a Leica SP5-AOBS Confocal Laser Scanning Microscope attached to a Leica DM I6000 inverted Epifluorescence Microscope with a 63X glycerol lens. Movies were taken at 60s/frame and were exported from Volocity® 5.3.1 as Quick time movies using the Sornson3 video compressor at 6 frame/second.

Time lapse movie quantification, cell tracking and cell speed measurements
Time lapse movie tracking of individual LysC:DsRed$^+$ cells was done using Image J 1.4, manual tracking plugin. LysC:DsRed$^+$ cell migration velocity was calculated using tracking object command in Volocity 5.3.1 measurement module (Perkin Elmer-Improvision, UK).

References

1. Cha, Y.I., Kim, S.H., Sepich, D., Buchanan, F.G., Solnica-Krezel, L., and DuBois, R.N. (2006). Cyclooxygenase-1-derived PGE2 promotes cell motility via the G-protein-coupled EP4 receptor during vertebrate gastrulation. Genes Dev *20*, 77-86.
2. Rhodes, J., Hagen, A., Hsu, K., Deng, M., Liu, T.X., Look, A.T., and Kanki, J.P. (2005). Interplay of pu.1 and gata1 determines myelo-erythroid progenitor cell fate in zebrafish. Dev Cell *8*, 97-108.
3. Liongue, C., Hall, C.J., O'Connell, B.A., Crosier, P., and Ward, A.C. (2009). Zebrafish granulocyte colony-stimulating factor receptor signaling promotes myelopoiesis and myeloid cell migration. Blood *113*, 2535-2546.
4. Li, L., Jin, H., Xu, J., Shi, Y., and Wen, Z. (2011). Irf8 regulates macrophage versus neutrophil fate during zebrafish primitive myelopoiesis. Blood *117*, 1359-1369.
