# Supplementary figures and images for: Live Imaging of Tumor Initiation in Zebrafish Larvae Reveals a Trophic Role for Leukocyte-Derived PGE2
Source: Curr Biol. 2012 Jul 10;22(13):1253–9. doi: 10.1016/j.cub.2012.05.010 (PMC3398414; doi:10.1016/j.cub.2012.05.010)

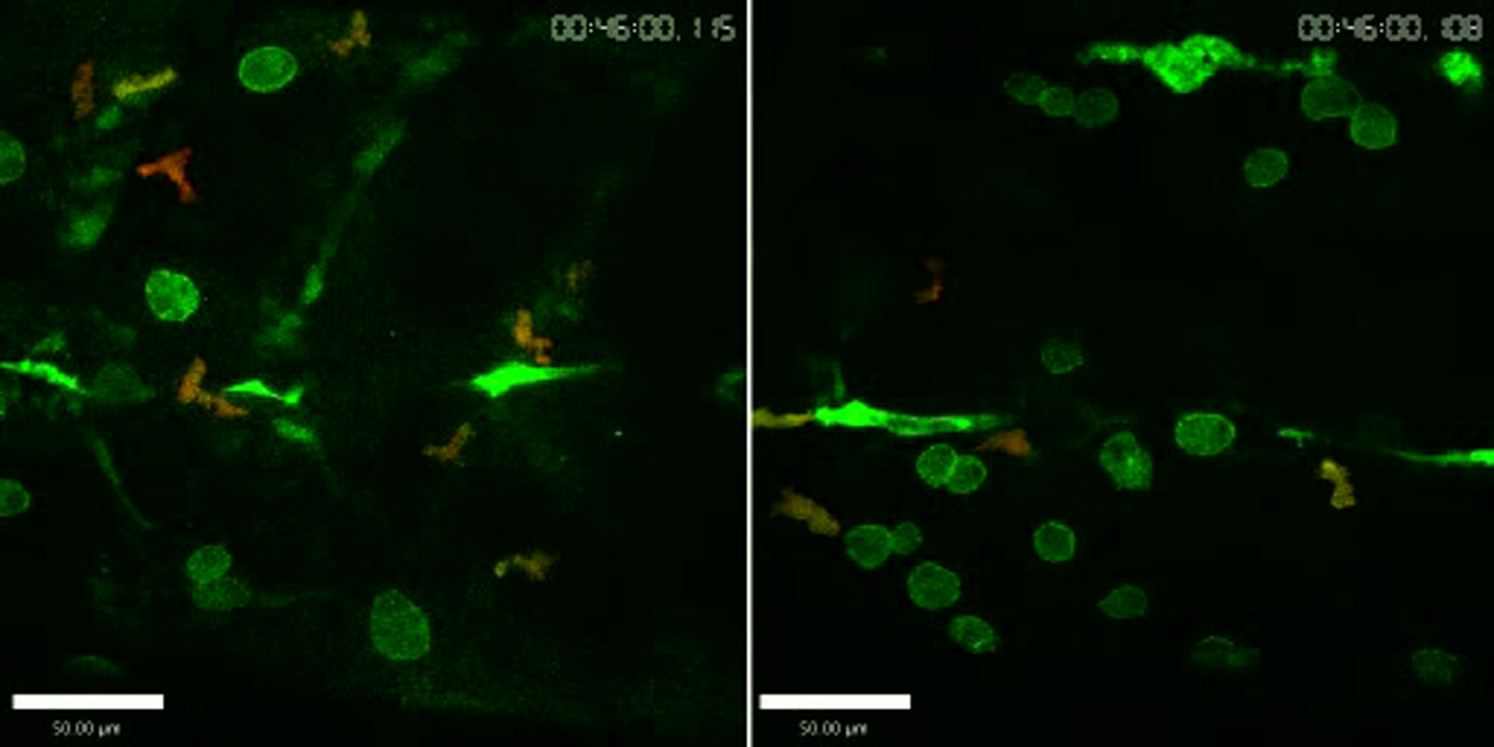

Supplement: Movie S1. Supporting Movies for Figures 4A and 4B — Movies from the flank skin region of a Tg(kit a:GalTA4, UAS:V12RASeGFP, lysC:DsRed) larva at 3 dpf. Green cells are V12RAS+ transformed mucus-secreting cells; red cells are LysC:DsRed labeled neutrophils. On the left is a typical 3 dpf control larva where we see LysC:DsRed+ leukocytes actively patrolling through the territory occupied by transformed cells. On the right is a typical movie of an NS398-treated larva showing significantly reduced neutrophil patrolling velocity at 3 dpf. [file mmc2.jpg]

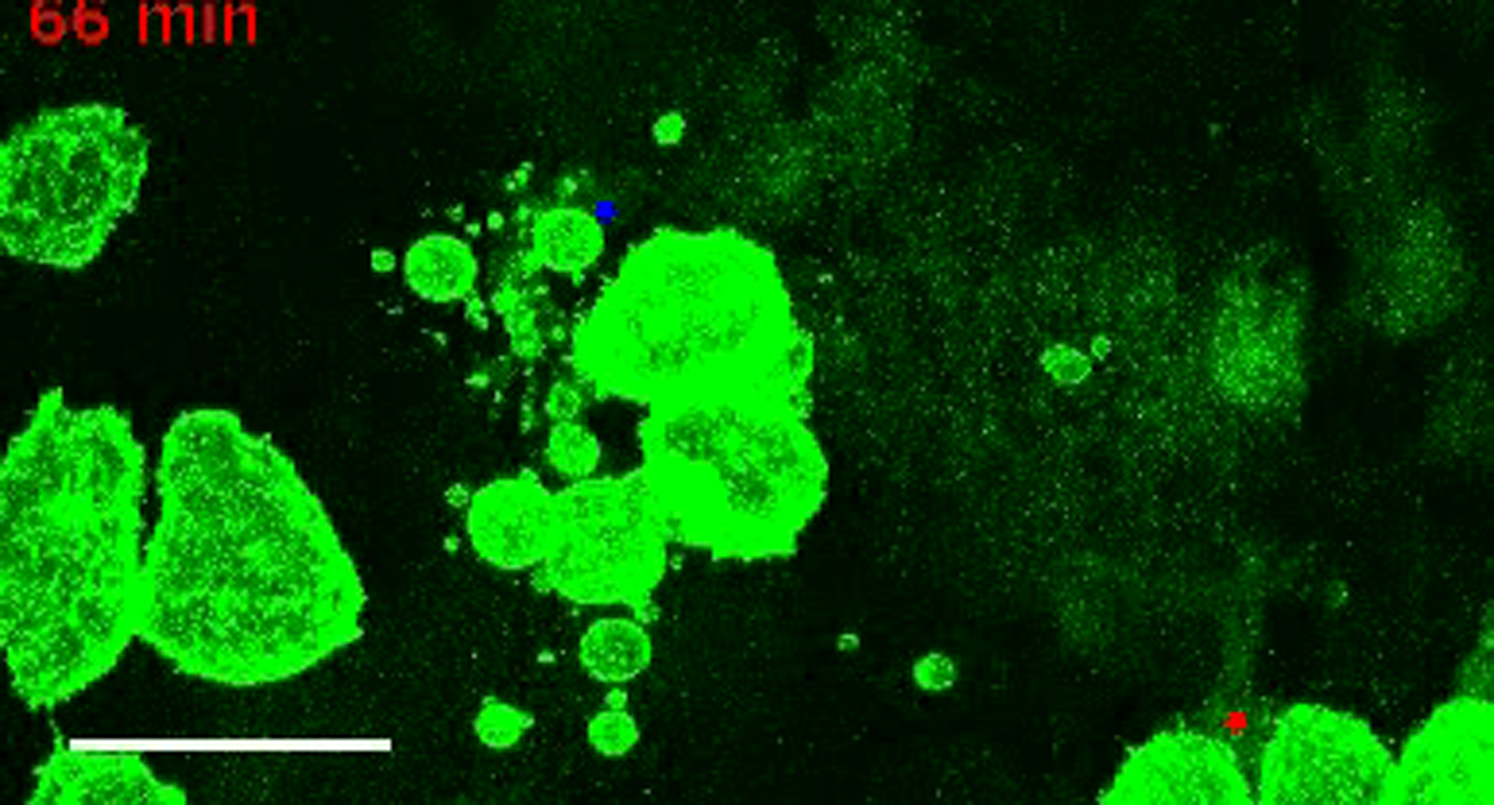

Supplement: Movie S2. Supporting Movie for Figure 4D — Movie from the flank skin region of a typical Tg(kit a:GalTA4, UAS:V12RASeGFP, NFκB-RE:eGFP) larva treated with NS398. Rounded bright green cells are V12RAS+ transformed mucus-secreting cells, and elongated pale green cells are patrolling macrophages. A red arrow indicates an early macrophage (blue dot) as it arrives in the vicinity of the clone of transformed cells and makes initial direct contacts with one of the cells in the group. Soon afterward, the transformed cell undergoes a catastrophic cell death, and the first macrophage as well as others (purple arrow, red dot) are seen engulfing debris resulting from this death. [file mmc3.jpg]
